# Antibody-dependent cellular cytotoxicity responses and susceptibility influence HIV-1 mother-to-child transmission

**DOI:** 10.1172/jci.insight.159435

**Published:** 2022-05-09

**Authors:** Allison S. Thomas, Carolyn Coote, Yvetane Moreau, John E. Isaac, Alexander C. Ewing, Athena P. Kourtis, Manish Sagar

**Affiliations:** 1 Department of Microbiology, Boston University School of Medicine, Boston, Massachusetts, USA.; 2 Department of Medicine, Boston Medical Center, Boston, Massachusetts, USA.; 3 Division of HIV Prevention, Centers for Disease Control and Prevention, Atlanta, Georgia, USA.

**Keywords:** AIDS/HIV, Infectious disease, AIDS vaccine, Adaptive immunity

## Abstract

HIV-1 vaccine efforts are primarily directed toward eliciting neutralizing antibodies (nAbs). However, vaccine trials and mother-to-child natural history cohort investigations indicate that antibody-dependent cellular cytotoxicity (ADCC), not nAbs, correlate with prevention. The ADCC characteristics associated with lack of HIV-1 acquisition remain unclear. Here, we examine ADCC and nAb properties in pretransmission plasma from HIV-1–exposed infants and from the corresponding transmitting and nontransmitting mothers’ breast milk and plasma. Breadth and potency (BP) were assessed against a panel of heterologous, nonmaternal variants. ADCC and neutralization sensitivity were estimated for the strains in the infected mothers. Infants who eventually acquired HIV-1 and those who remained uninfected had similar pretransmission ADCC_BP_. Viruses circulating in the transmitting and nontransmitting mothers had similar ADCC susceptibility. Infants with higher pretransmission ADCC_BP_ and exposure to more ADCC-susceptible strains were less likely to acquire HIV-1. In contrast, higher preexisting infant neutralization BP and greater maternal virus neutralization sensitivity did not associate with transmission. Infants had higher ADCC_BP_ closer to birth and in the presence of high plasma IgG relative to IgA levels. Mothers with potent humoral responses against their autologous viruses harbored more ADCC-sensitive strains. ADCC sensitivity of the exposure variants and preexisting ADCC_BP_ influenced mother-to-child HIV-1 transmission during breastfeeding. Vaccination strategies that enhance ADCC are likely insufficient to prevent HIV-1 transmission because some strains may have low ADCC susceptibility.

## Graphical abstract

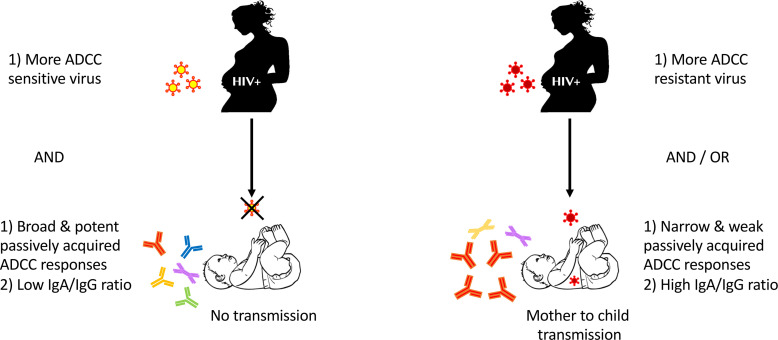

## Introduction

Developing an effective HIV-1 vaccine remains a top priority. In general, all vaccines work by generating Abs that block infection or interfere with viral replication (1). In the case of HIV-1, neutralizing Abs (nAbs) are insufficient to prevent infection. Recent large clinical trials demonstrated that passive infusion of large quantities of a broadly neutralizing Ab (bnAb) did not significantly reduce HIV-1 transmission, presumably because individuals are commonly exposed to neutralization-resistant strains (2). Chronically infected mothers who expose their babies to HIV-1 during gestation, delivery, or breastfeeding also harbor mostly variants that are resistant to neutralization by the Abs present in their plasma and breast milk (BM) and the Abs acquired by the infant (3–6). Thus, in the presence of preexisting Abs, most human transmission occurs with neutralization-resistant viruses. The passive infusion of more than 1 bnAb may significantly reduce HIV-1 transmission because it may be able to block the majority of neutralization-resistant viruses, but this has not been confirmed in human clinical trials.

Ab-dependent cellular cytotoxicity (ADCC), potentially along with other Ab-mediated effector functions, may protect against infection in instances where preexisting nAbs cannot block neutralization-resistant strains. ADCC requires Ab Fab domain binding to the HIV-1 envelope (Env) on infected cells. Fc engagement with Fc receptors (FcRs), such as FcγRIIIa (CD16) on NK cells, induces effector cells to kill the infected cell (7). We have recently shown that ADCC responses against the variants circulating in infected mothers are significantly higher in breastfed infants who did not acquire HIV-1 (HIV-1–exposed uninfected, HEU) as compared with those who eventually acquired infection (HIV-exposed infected, HEI) (8). Furthermore, our studies confirmed some previous findings that infected infants with higher ADCC responses have lower morbidity and mortality over the first year after birth in the absence of antiretroviral therapy (ART; refs. 9, 10). In aggregate, these observations suggest that enhancing ADCC responses may both protect against the acquisition of neutralization-resistant strains and improve disease outcomes in infants who are infected.

In our previous study, we assessed pretransmission ADCC responses present in the exposed infants against the variants circulating in their own mothers. Observing that HEU as compared with HEI infants have higher ADCC specifically against their mothers’ strains suggests multiple nonmutually exclusive possibilities. It is possible that HEU as compared with HEI infants have broader and more potent ADCC responses against all viruses, both the strains circulating in the infected mother and unrelated heterologous variants. This finding would potentially be important because future vaccines may be able to enhance overall ADCC responses against all HIV-1 strains rather than just those present in the transmission source. Another possibility is that HEU compared to HEI infants have similar ADCC breadth and potency (ADCC_BP_), but the nontransmitting mothers (NTMs) have strains that are more susceptible to ADCC when compared with the transmitting mothers (TMs). This observation would imply that transmission efficacy depends more on the characteristics of the variants present in the transmitting partner rather than the preexisting responses in the exposed individual. It would be difficult to overcome this mechanism with a future vaccine. Finally, a combination of more potent ADCC activity and exposure to less ADCC-resistant virus strains may differentiate HEI and HEU infants.

Here, we show that infants with a combination of greater pretransmission ADCC along with exposure to more ADCC-susceptible stains are less likely to acquire HIV-1. HEI and HEU infants, however, have similar ADCC_BP_, and TMs and NTMs harbor strains with similar ADCC sensitivity. Enhancing ADCC may not prevent infection if circulating strains are mostly ADCC resistant. Efforts to eliminate HIV-1 transmission will need to both improve ADCC responses in at-risk individuals and account for the ADCC susceptibility of the strains circulating in the infected individuals most likely to transmit the virus.

## Results

### ADCC_BP_ is not different among TM- and NTM-infant pairs.

**Figure 1:**
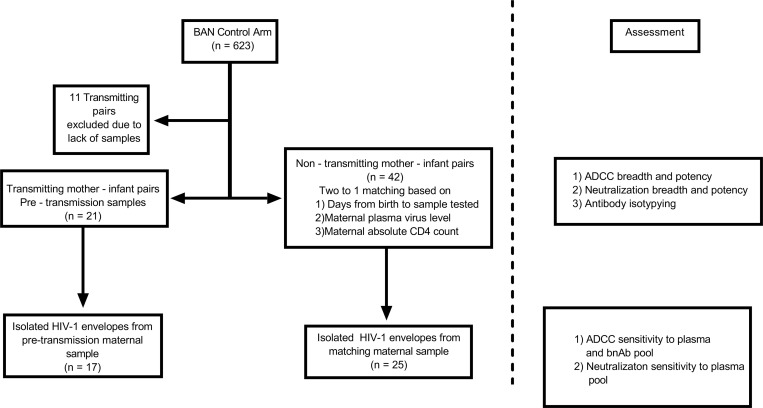
Study design. Flow diagram of TM-and matching NTM-infant pairs from the BAN study control arm. Matching was based on the specified criteria. Right side of figure shows the assessment on the samples at each level.

We examined ADCC responses in plasma and BM samples from mother-infant pairs in the control arm of the Breastfeeding, Antiretroviral, and Nutrition (BAN) study (Figure 1; ref. 11). Dyads were enrolled in this trial after confirming the infants had no evidence of HIV-1 infection within the first 14 days after birth. The mothers and infants in the control arm received ART for 7 days postpartum and not prior to delivery. Pretransmission responses were assessed in the closest available sample collected before the first infant HIV-1 PCR-positive result. A total of 21 TM-infant pairs were matched to 42 pairs with no documented transmission based on maternal age, plasma virus level, absolute CD4^+^ T cell count, and duration of time from birth to sample collection (Supplemental Table 1; supplemental material available online with this article; https://doi.org/10.1172/jci.insight.159435DS1). We quantified ADCC_BP_ by assessing responses against 10 HIV-1 Envs of diverse clades. These 10 highly divergent Envs, unrelated to the individuals in this cohort, are part of a global reference panel that has been previously used to estimate the neutralization spectrum of infected and vaccinated individuals (12). ADCC was estimated using a previously described assay that quantifies HIV-1–infected cell killing only in the presence of plasma or isolated IgG (13). An ADCC_BP_ score was derived using previously detailed methods (14, 15). Briefly, ADCC_BP_ consists of the average of the log-normalized percentage ADCC at 1 tested plasma concentration against the 10 Envs. Importantly, the percentage decrease at the highest tested plasma dilution (1:50) strongly correlated with both the dilution required to achieve ID_50_ and AUC (Supplemental Figure 1; refs. 14, 16). ADCC_BP_ scores ranged from a minimum of 0 indicating no ADCC capacity, to 1, which represents 100% killing against all 10 tested Envs.

**Figure 2:**
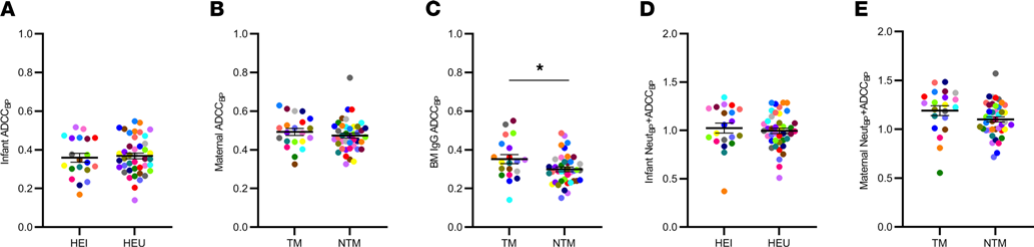
Infant and maternal ADCC_BP_ are similar. The following are shown in relation to transmission outcomes: (**A**) ADCC_BP_ in HEI and HEU infants; (**B** and **C**) ADCC_BP_ in TMs and NTMs and IgG isolated from BM; (**D**) ADCC_BP_ plus Neut_BP_ in HEI and HEU infants; and (**E**) ADCC_BP_ plus Neut_BP_ in TMs and NTMs. Colors signify matched pairs. Bars show mean and standard error. Group comparisons were done using Welch’s *t* test and/or multivariable logistic regression. *P ≤ 0.05 with 1 of these statistical tests. All values are means from a minimum of 2 replicates.

HEU (mean 0.359, range 0.168–0.517) compared to HEI (mean 0.368, range 0.140–0.548) infants had similar ADCC_BP_ (*P* *=* 0.740; Figure 2A). Neither was ADCC_BP_ different in TM (mean 0.489, range 0.326–0.629) or NTM (mean = 0.468, range 0.326–0.773) plasma (*P* = 0.396; Figure 2B). ADCC_BP_ trended higher in TM (mean 0.338, range 0.142–0.551) than in NTM (mean 0.290, range 0.151–0.487) BM IgG (*P* = 0.055; Figure 2C). Multivariable logistic regression analysis demonstrated that the odds of a mother transmitting the virus to the baby were approximately 2-fold higher with a 0.1-unit increase in BM IgG ADCC_BP_ (OR 2.10, 95% CI 1.08–4.48, *P* = 0.037) after accounting for maternal plasma virus level, absolute CD4^+^ T cell count, and duration between birth and sample collection. In contrast to the infant and maternal plasma, BM IgG ADCC was significantly higher in 3 of the 10 Envs in TMs compared with NTMs (Supplemental Figure 2).

We have also previously estimated neutralization BP (Neut_BP_) in the same infant and maternal plasma samples by measuring neutralization capacity against the same reference Env panel (14). In our prior work, we also found that a combination of higher infant ADCC plus nAb responses against the maternal autologous strains (Neut_AUC_ + ADCC_AUC_) are associated with lower HIV-1 acquisition (8). In a similar manner, we summed the 2 BP scores in our current study to generate an overall aggregate statistic (Neut_BP_ + ADCC_BP_) that represents multiple Ab functions. HEI (mean 1.01, range 0.370–1.34) compared to HEU (mean 0.993, range 0.508–1.29, *P* = 0.630) infants and TMs (mean 1.25, range 0.555–1.48) versus NTMs (mean 1.09, range 0.715–1.57, *P* = 0.123) did not have significantly different Neut_BP_ + ADCC_BP_ (Figure 2, D and E).

### TMs and NTMs harbor variants with similar ADCC susceptibility.

**Figure 3:**
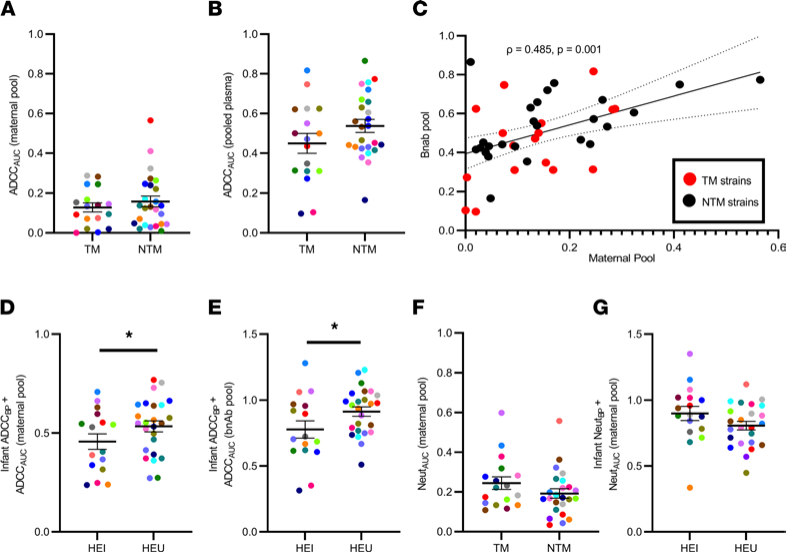
Infant ADCC responses and ADCC sensitivity of the exposure strains associate with transmission. ADCC sensitivity of TM and NTM viruses to (**A**) pool of maternal plasma and (**B**) bnAbs. (**C**) ADCC susceptibility correlation to maternal pool and bnAbs. Combined statistic of infant ADCC_BP_ and sensitivity of TM and NTM strains to (**D**) maternal pool — ADCC_AUC_ _(maternal_ _pool)_ — or (**E**) bnAbs — ADCC_AUC_ _(bnAb_ _pool)_ — among HEI and HEU infants. (**F**) Neutralization susceptibility of TM and NTM Envs to maternal plasma pool and (**G**) combined statistic of infant Neut_BP_ and neutralization susceptibility of the exposure strains. Colors signify matched pairs. Bars show mean and standard error. Group comparisons were done using Wilcoxon’s rank-sum test for **A** and **B**, Welch’s *t* test for **D**–**G**, and/or multivariable logistic regression for **D**, **E**, and **G**. Correlations were assessed using Spearman’s statistic. *P ≤ 0.05 with 1 of these statistical tests. All values are means from a minimum of 2 replicates.

While HEU compared to HEI infants had similar ADCC_BP_ against heterologous variants (Figure 2), they had higher ADCC against their corresponding mothers’ strains (8). Thus, we next hypothesized that the ADCC susceptibility of viruses from NTMs is higher than those from TMs. To address this hypothesis, we examined the ADCC sensitivity of viruses generated in our previous mother-to-child transmission (MTCT) studies, namely those that incorporated Envs from 17 TMs and 25 NTMs (Figure 1; refs. 8, 14). Susceptibility was assessed against 2 common standards: a maternal plasma pool and a bnAb pool. The maternal plasma pool contained an equivalent amount of plasma from 10 different NTMs enrolled in the BAN study who were distinct from the 25 NTMs used to generate the maternal variants. The bnAb pool contained an equivalent amount of 4 bnAbs (VRC01, PGT121, PG16, and 10E8) (17–20). ADCC over a series of plasma/Ab dilutions was used to generate an AUC. A higher AUC indicates greater ADCC sensitivity and vice versa. NTM compared with TM variants had similar AUC against both the maternal pool (mean 0.158, range 0.010–0.565 versus mean 0.128, range 0.0–0.287, *P* = 0.648; Figure 3A) and the bnAbs (mean 0.538, range 0.166–0.866 versus mean 0.450, range 0.09–0.817, *P* = 0.176; Figure 3B). AUCs against the maternal and bnAb pool were modestly correlated for the 42 different maternal viruses (Figure 3C).

### Combination of infant ADCC_BP_ and maternal variant ADCC susceptibility is associated with transmission.

Next, we hypothesized that a combination of preexisting ADCC responses and susceptibility of the exposure strains influence transmission. We generated a combined metric for pretransmission ADCC activity and exposure variant susceptibility by adding infant ADCC_BP_ and the ADCC_AUC_ _(maternal_ _pool)_ or ADCC_AUC_ _(bnAb_ _pool)_ of the corresponding mother’s strains. It should be noted that ADCC_BP_ and ADCC_AUC_ are independent because they are generated against different viruses. A higher magnitude of this metric implies a combination of increased ADCC_BP_ and exposure to more ADCC-sensitive strains. HEU (mean 0.534, range 0.272–0.770) had higher ADCC_BP_ + ADCC_AUC_ _(maternal_ _pool)_ compared with HEI (mean 0.457, range 0.237–0.709, *P* = 0.110; Figure 3D) infants. ADCC_BP_ + ADCC_AUC_ _(bnAb_ _pool)_ was also higher in HEU (mean 0.961, range 0.510–1.23) as opposed to HEI (mean 0.778, range 0.314–1.28, *P* = 0.046; Figure 3E) infants. In multivariate logistic regression analysis that accounted for baseline maternal characteristics and days from birth to sample collection, a 0.1-unit increase in ADCC_BP_ + ADCC_AUC_ _(maternal_ _pool)_ (OR 0.604, 95% CI 0.356–0.937, *P* = 0.037) and ADCC_BP_ + ADCC_AUC_ _(bnAb_ _pool)_ (OR 0.648, 95% CI 0.397–0.974, *P* = 0.048) was associated with an approximately 40% and 35% reduction of HIV-1 acquisition, respectively.

We next examined if transmission was also lower among infants who had higher preexisting Neut_BP_ while being exposed to more neutralization-sensitive strains from their corresponding mothers. Infant pretransmission Neut_BP_ was estimated previously (14). We assessed neutralization sensitivity of the virus stocks incorporating Envs from the infected mothers against the same maternal plasma pool used previously. As before, higher AUC indicates greater neutralization sensitivity and vice versa. Neutralization AUCs could not be generated against the bnAb pool because all virus stocks were exquisitely sensitive at the concentrations tested previously. Similar to previous reports (3–6), TM and NTM Envs had similar neutralization susceptibility (Figure 3F). A combination of pretransmission Neut_BP_ in the exposed infant and the neutralization sensitivity of the corresponding mother’s strain, Neut_BP_ + Neut_AUC_ _(maternal_ _pool)_, trended higher in the transmitting (mean 0.899, range 0.336–1.35) as compared with the nontransmitting (mean 0.807, range 0.448–1.12, *P* = 0.083, Figure 3G) dyads. In multivariate logistic regression analysis that accounted for baseline maternal plasma virus level, absolute CD4^+^ T cell count, and days from birth to sample collection, a 0.1-unit increase in Neut_BP_ + Neut_AUC_ associated with around 36% higher transmission (OR 1.36, 95% CI 0.946–2.13, *P* = 0.123), although this was not statistically significant. Thus, in contrast to ADCC, higher infant pretransmission Neut_BP_ and exposure to more neutralization-sensitive maternal viruses was not associated with decreased likelihood of HIV-1 acquisition.

### Infant ADCC responses are associated with IgG/IgA, days from birth, and neutralization.

**Figure 4:**
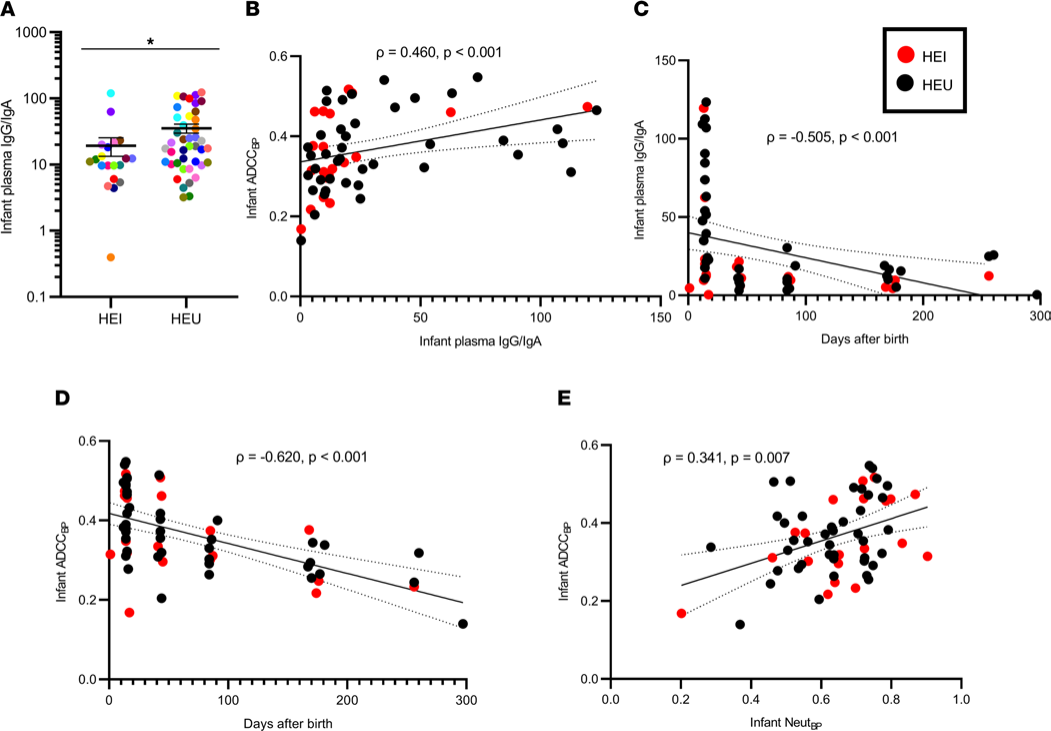
Predictors for infant plasma IgG/IgA and ADCC_BP_. (**A**) Infant IgG/IgA ratio by transmission status. Colors signify matched pairs. Bars show mean and standard error. Comparison was done using Wilcoxon’s rank-sum test; *P *≤* 0.05. (**B**–**E**) Correlations among the variables identified in the *x* and *y* axis. The red and black dots indicate HEI and HEU infants, respectively. Correlations were assessed using Spearman’s statistic. Line indicates linear regression fit with 95% CI. All values represent mean values from a minimum of 2 replicates.

We and others have shown that a high IgG/IgA ratio is associated with more effective ADCC because IgA may block the IgG-mediated Ab effector function (8, 21–23). In this larger cohort of 21 HEI/TMs and 42 HEU/NTMs, HEU (mean 35.36, range 3.21–120.2) compared with HEI (mean 19.32, range 0.397–119.5, *P* = 0.042) had a higher plasma IgG/IgA ratio (Figure 4A), and the IgG/IgA ratio was positively correlated with infant ADCC (Figure 4B). Infants acquire the majority of maternal IgG transplacentally during gestation, and after birth, babies ingest less IgG than IgA because BM contains high levels of IgA (8, 24). As expected, both the infant plasma IgG/IgA ratio (Figure 4C) and infant ADCC_BP_ (Figure 4D) decreased over time after birth. Importantly, in multivariable linear regression analysis, the infant IgG/IgA ratio and number of days from birth independently predicted infant ADCC_BP_ (Supplemental Table 1). In contrast, the IgG/IgA ratio was higher in NTM compared with TM plasma (Supplemental Figure 3A) but not in the BM (Supplemental Figure 3B), nor was it associated with the maternal ADCC_BP_ score (Supplemental Figure 3, C and D).

Previous analyses from our group and others have suggested that ADCC and neutralization are independent activities (8, 13, 25, 26). Maternal ADCC_BP_ did not correlate with the Neut_BP_ score (Supplemental Figure 3E). Neither did maternal ADCC_BP_ correlate with the plasma virus load or absolute CD4^+^ T cell count (Supplemental Figure 3, F and G). In contrast, infant ADCC_BP_ showed a modest association with the Neut_BP_ score (Figure 4E).

### ADCC susceptibility of the maternal variants is associated with days from birth and humoral capacity.

**Figure 5:**
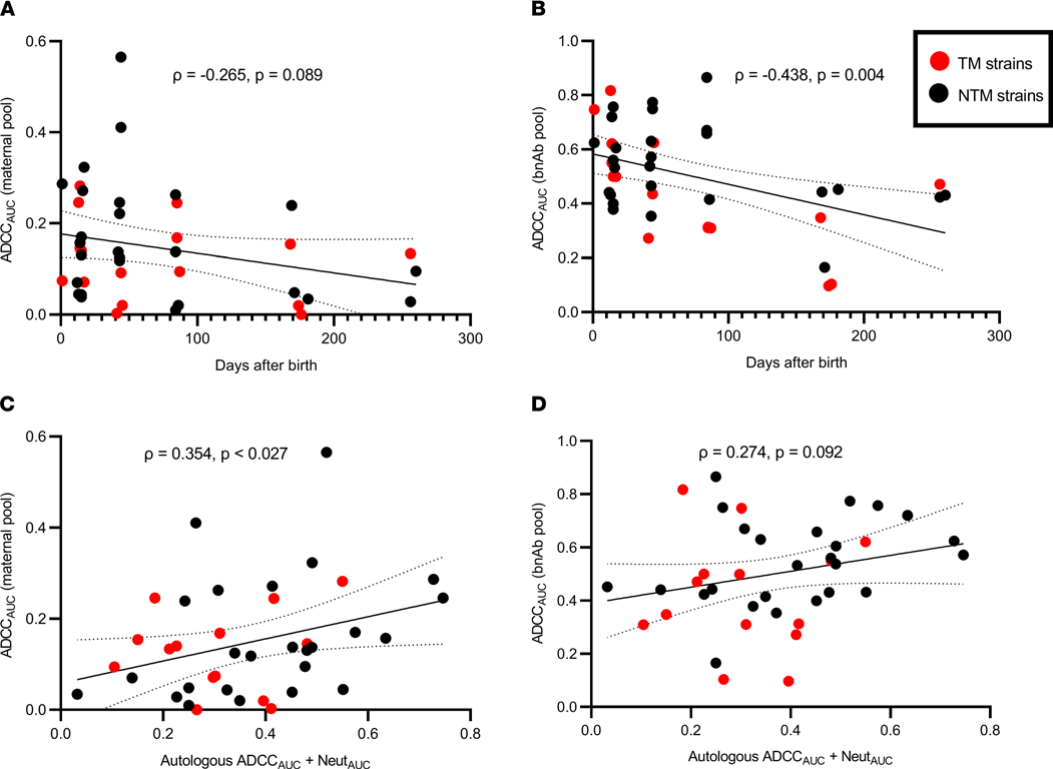
Predictors of ADCC susceptibility of the maternal variants. Correlation between maternal variant ADCC susceptibility to (**A** and **C**) pool of mother plasma and to (**B** and **D**) bnAbs. Associations are with days from birth in **A** and **B** and with Neut_AUC_ + ADCC_AUC_ score in **C** and **D**. The red and black dots indicate TMs and NTMs, respectively. Correlations were assessed using Spearman’s statistic. Line indicates linear regression fit with 95% CI.

Factors associated with ADCC susceptibility have not been identified. In general, it is well known that variants circulating in infected hosts become more neutralization resistant over time as they evolve to escape autologous nAbs (27–29). In a similar fashion, ADCC susceptibility to both the plasma pooled from different mothers (Figure 5A) and bnAbs (Figure 5B) of the maternal variants decreased with the increasing sampling date relative to birth. Furthermore, the investigation of a small number of Env variants has suggested that nAb selection pressure can increase sensitivity to ADCC (30, 31). We, however, did not observe any association between ADCC susceptibility, to either maternal or bnAb pool, with the neutralization capacity or ADCC in the contemporaneous autologous maternal plasma or with the maternal neutralization or ADCC_BP_ score (ρ < 0.2 and *P* *>* 0.05 in all cases, data not shown). ADCC susceptibility, however, increased with higher levels of the previously described statistic, Neut_AUC_ + ADCC_AUC_, which estimates the combined neutralization and ADCC present in the contemporaneous plasma against the autologous circulating strain (Figure 5, C and D, and ref. 8).

### Infant morbidity and mortality are not associated with ADCC_BP_.

**Figure 6:**
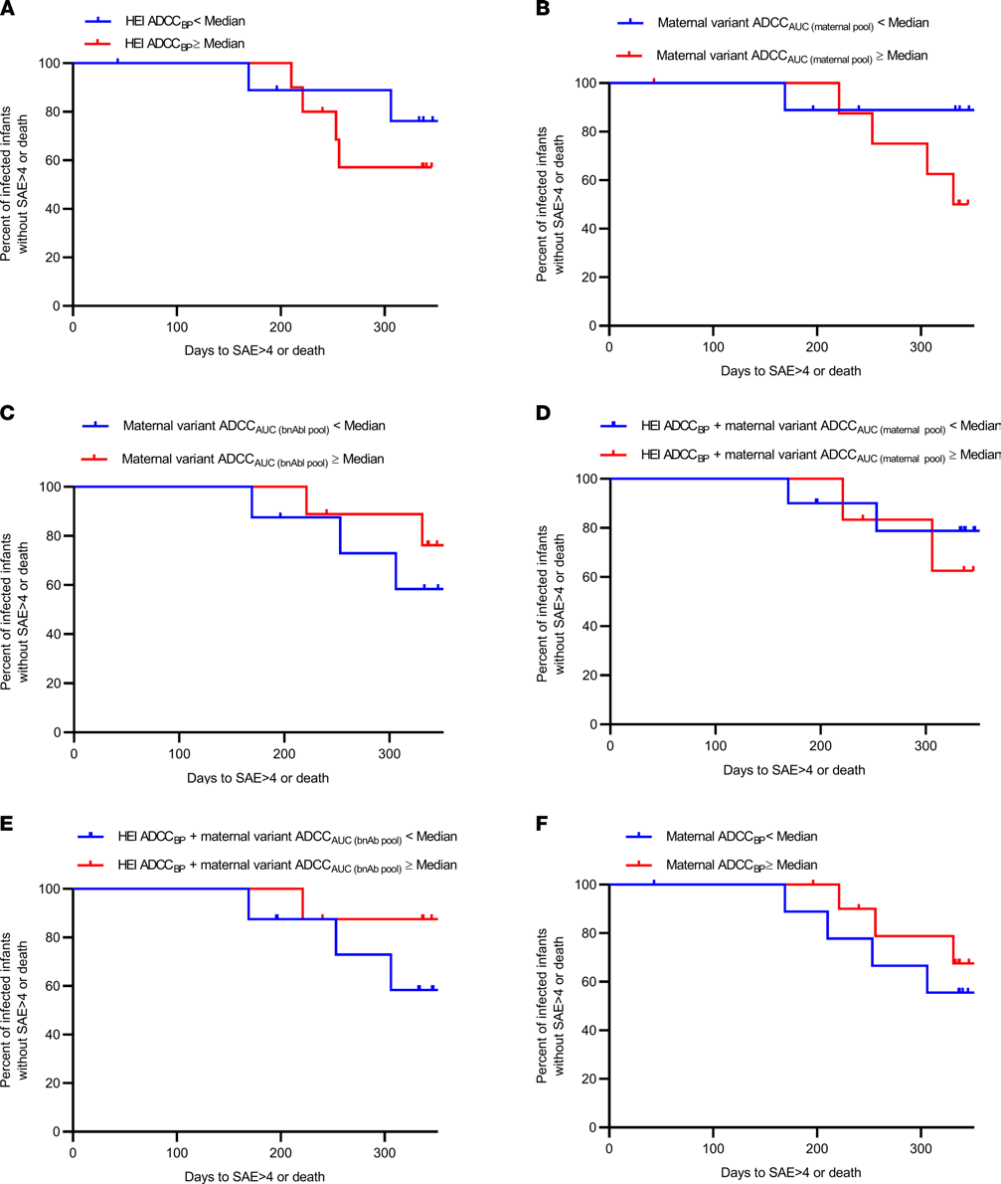
Morbidity associated with preexisting humoral responses. Kaplan-Meier curves estimating time (days) to a grade 4 or greater serious adverse event or death for (**A**–**E**) HEI and (**F**) all infants with different measures greater than or equal to the cohort median (red) or less than the cohort median (blue). Tick marks denote right censoring.

Work from our group suggested that infected infants with higher ADCC against their mother’s virus had decreased morbidity and mortality up to 1 year after birth in the absence of ART (8). Other investigations suggest that higher infant ADCC against variants that are different but similar to the corresponding mother’s strains also associates with better outcomes (9, 10). Here, we found that infant morbidity and mortality up to 1 year after birth were not associated with infant ADCC_BP_ (Figure 6A), ADCC susceptibility of the exposure strains (Figure 6, B and C), or a combination of infant ADCC_BP_ and sensitivity of the exposure variants (Figure 6, D and E). Neither were infant outcomes significantly different in relation to ADCC against any of the individual 10 heterologous Envs used to derive the ADCC_BP_ score (data not shown). Infants born to mothers with ADCC_BP_ greater than the cohort median had lower morbidity and mortality up to 1 year after birth, although this was also not statistically significant (Figure 6F).

## Discussion

Recent efforts aimed at developing an HIV-1 vaccine have focused on eliciting nAbs. However, 2 large randomized clinical trials demonstrated that the presence of high levels of a bnAb did not decrease HIV-1 transmission (2). Similarly, we and others have demonstrated that some infants born to mothers with HIV-1 have broad and potent nAbs soon after birth, but they still acquire HIV-1 from their mother within the first year of their lives (14, 32). In aggregate, nAbs are insufficient to prevent transmission primarily because naive individuals are exposed to a viral swarm that may contain neutralization-resistant variants. However, MTCT cohorts and vaccine trial correlative analyses suggest that preexisting ADCC alone, and potentially in conjunction with nAbs, can decrease the transmission of these neutralization-resistant strains (8, 9, 21, 33–35). In these prior studies, ADCC was either examined against the viruses circulating in the transmission source or a small number of Envs that were highly related to the potentially exposure variants. These results possibly imply that ADCC efficacy depends on the ability to specifically target Envs in the exposure material. It remains uncertain if preexisting ADCC responses that potently target a diverse range of unrelated HIV-1 variants is associated with protection.

In this study, we estimated ADCC_BP_ by examining activity against 10 unrelated, highly divergent, HIV-1 Envs. These 10 Envs encompass 7 different HIV-1 clades, which differ by a minimum of 10% at a nucleotide level (12, 36). Thus, the 10 Envs represent a broad swath of the circulating variants that may need to be targeted to prevent HIV-1 acquisition in high-risk individuals around the world. We developed a potentially novel ADCC_BP_ score to quantify responses against the unrelated strains present in HIV-1–exposed infants and their chronically infected mothers. We find that HEI and HEU infants and TMs and NTMs had similar ADCC_BP_ in their plasma. Our results are consistent with a previous study that examined ADCC activity in a smaller number of maternal samples only from the same BAN cohort against 1 heterologous Env (37). Our observations imply that preexisting broad and potent ADCC activity in the exposed or the transmitting individual does not associate with transmission outcome.

We further demonstrate that ADCC sensitivity of the variants circulating in the TMs and NTMs was not significantly different. We assessed ADCC susceptibility against 2 Ab standards: a maternal plasma pool and a bnAb pool. The ADCC sensitivity to these 2 different Ab standards was moderately correlated, suggesting that they measured a similar virus property. Similar ADCC susceptibility among the variants from TMs and NTMs suggests that the observation of higher ADCC against their corresponding mother’s viruses among HEU compared with HEI infants cannot be explained by exposure to more susceptible strains. Importantly, we observed that HEU compared with HEI infants had a higher magnitude of a metric that combined ADCC_BP_ and ADCC sensitivity of the exposure strains. On the other hand, a combined statistic of Neut_BP_ and neutralization susceptibility of the maternal variants was not different among TM- as compared with NTM-infant pairs. In aggregate, our findings suggest that both the magnitude and breadth of preexisting ADCC, more than neutralizing activity, and the ADCC sensitivity, more than neutralization susceptibility, of the strains present in the transmission source are important for preventing the acquisition of neutralization-resistant strains.

Our findings potentially provide insights into the divergent outcomes between 2 different recent HIV-1 vaccine trials (38, 39). Both trials used a relatively similar heterologous prime/boost regimen (40). The modestly effective RV144 trial used a clade B/E Env immunogen tailored to the viruses circulating in Thailand. On the other hand, the HVTN 702 trial, which was stopped early because of lack of efficacy, used a HIV-1 subtype C Env immunogen better suited for the strains prevalent in Africa. Both vaccine products generated high levels of ADCC activity, which was associated with protection in the RV144 correlative analysis (21). The 2 trials may have produced different outcomes because of differences in the strains circulating in Thailand as opposed to those in Africa. A prior study has suggested that a majority of viruses commonly found in Thailand contain a sequence polymorphism that renders Envs highly ADCC susceptible (41). Strains in Africa and other parts of the world do not have this Env change. Differences in ADCC sensitivity among the majority of strains circulating in Thailand, as compared with Africa, may be a reason for the different outcomes in the 2 vaccine trials even though both test products generated high ADCC levels.

The ADCC characteristics of the exposure strains are also important for the morbidity and mortality after infection in the absence of ART. We have previously shown that infected infants with higher ADCC activity against their corresponding mother’s circulating viruses have lower morbidity and mortality up to 1 year after birth without ART (8). Here we found that better outcomes were not observed in the infected infants with higher ADCC_BP_ exposure to more sensitive maternal strains or a combination of the 2 metrics. This argues that infant ADCC responses need to effectively target the infecting strains to influence subsequent morbidity and mortality. Our findings suggest that ADCC_BP_, as quantified by measuring responses against unrelated heterologous Envs, does not adequately reflect the activity against the viruses actually present in the infected infants.

ADCC_BP_ may be improved by decreasing IgA proportions in the exposed individual because higher a IgG/IgA ratio was associated with stronger ADCC activity. Our observations in this study confirm previous suggestions that IgA inhibits IgG-mediated ADCC (8, 21–23). The higher IgG/IgA levels in HEU compared with HEI infants do not merely reflect higher IgG/IgA BM levels in the NTMs as opposed to TMs. Although the NTMs as compared with TMs had higher plasma IgG/IgA, they did not have higher IgG/IgA in the BM. Surprisingly, we observed that BM IgG–mediated ADCC_BP_ was higher in TMs than in NTMs. This finding is somewhat analogous to our previous observation that broader and more potent maternal plasma nAbs responses tracked with transmission (14). The HEI as compared with HEU infants do not have higher ADCC_BP_, implying that the broader and more potent IgGs present in the TM BM are not acquired by the breastfed infant. Previous investigations have shown that infants selectively acquire specific Abs from their mothers during breastfeeding, and this selective transfer is influenced by disease states (42, 43). Furthermore, BM contains much higher levels of IgA relative to plasma, and this could potentially explain the decrease in infant ADCC_BP_ after birth even though they continue to ingest maternal Abs via breastfeeding. Even though we did not measure HIV-1–specific Ig and isotype levels, our findings suggest that total plasma IgA levels in exposed individuals hinder ADCC activity.

In contrast to our prior studies, we observed that exposed infants’ neutralization and ADCC_BP_ were highly correlated (8, 13). In general, ADCC and neutralization are independent activities (25, 26). This correlation observed in the infants may again reflect the preferential transfer of ADCC-mediating IgG from the mother to the baby (42, 43). Collectively, our observations suggest that infants that selectively acquire both higher amounts of maternal IgG relative to IgA and Abs with the ability to mediate cellular cytotoxicity are more likely to resist HIV-1 acquisition.

In contrast to neutralization, studies have not identified host characteristics associated with the presence of ADCC-sensitive strains. In general, naive individuals that do not have preexisting nAbs mostly acquire neutralization-sensitive strains (44). Infected individuals develop Abs against the infecting viruses, but the autologous variants continue to evolve and escape this immune pressure (27–29). In general, individuals with longer duration of infection and more potent nAbs have more neutralization-resistant strains (45, 46). We observed that the maternal variants isolated closer to the birth of the child were more ADCC sensitive compared with those isolated after delivery within the first year of the infant’s life. This potentially also reflects increasing ADCC resistance over time. On the other hand, some but not all consider pregnancy as an immune-altered state; thus, pregnancy may be associated with the generation of more ADCC-susceptible strains (47). We also found that maternal variant ADCC sensitivity correlated with the totality of the autologous neutralization and ADCC response against the contemporaneous strain. As suggested by a previous investigation, this may reflect Env evolution toward an ADCC-susceptible state in response to potent Ab responses (30). ADCC sensitivity of the maternal viruses, however, was not higher in those mothers with greater ADCC_BP_ or more ADCC against the autologous strain in the contemporaneous plasma (48). Our studies, however, were conducted in a cross-sectional cohort in a relatively small number of variants. Furthermore, we examined virus stocks that incorporated multiple and not individual maternal Envs. Thus, our studies cannot decipher individual Env features associated with ADCC sensitivity. It is possible that TMs and NTMs have differences in the proportion of ADCC-sensitive and -resistant Envs, though, in bulk, ADCC susceptibility may be similar. Examination of single genome amplified Envs from a larger number of individuals sampled longitudinally will be required to identify those factors that may influence the ADCC sensitivity of the circulating variants in chronically infected individuals.

Our results provide insights for HIV-1 vaccine efforts. To date, traditional vaccine methodologies have failed to elicit even 1 bnAb, and high levels of a single bnAb do not protect against HIV-1 acquisition (2, 49). This study, along with previous investigations in humans and animal models, suggests that ADCC may help prevent HIV-1 acquisition especially when nAbs are not sufficient (8, 21, 50). It remains unclear if Abs with the ability to mediate broad and potent cellular cytotoxicity can be elicited using current vaccine technologies. Our studies here, however, suggest that generating ADCC_BP_ will not be sufficient. Strategies will also need to account for the ADCC sensitivity of the circulating strains in individuals likely to transmit HIV-1.

## Methods

### Antibodies and cell cultures.

All bnAbs and HIVIG were obtained from the NIH AIDS Reagent Program. Melon Gel IgG Spin Purification Kit (Thermo Fisher Scientific, 45206) was used to isolate IgG from the BM supernatant per manufacturer’s protocol. All cells were maintained as previously described (8). Briefly, MT4-CCR5-Luc and CD16^+^KHYG-1 cells were maintained at a density of 1 × 10^6^ and 5 × 10^5^ cells/mL, respectively. Cells were in RPMI complete, which consists of RPMI 1640 (Invitrogen), 10% FBS (Invitrogen), and 25 mM HEPES (Invitrogen). MT4-CCR5-Luc media also contained 2 mM l-glutamine (Invitrogen), 100 U/mL penicillin (Invitrogen), and 100 μg/mL streptomycin (Invitrogen). The CD16^+^KHYG-1 media also contained 0.1 mg/mL Primocin (InvivoGen), 1 μg/mL Cyclosporine A (MilliporeSigma), and 10 U/mL IL-2 (NIH AIDS Reagent Program). Human epithelial kidney 293T (HEK293T) cells and TZM-bl cells were acquired from the NIH AIDS Reagent Program and maintained in DMEM containing 10% FBS (Invitrogen), 2 mM l-glutamine (Invitrogen), 100 U/mL penicillin (Invitrogen), and 100 μg/mL streptomycin (Invitrogen).

### Human participants and samples.

Plasma and BM samples were acquired from the control arm of the BAN study (ClinicalTrials.gov NCT00164736) as described previously (11). Each TM-infant pair was matched to dyads based on maternal plasma viral load, maternal CD4^+^ T cell count, and days postpartum to sample collection (14).

### Env isolation, amplification, and replication-competent virus stocks.

Maternal Envs were isolated, incorporated into an isogenic HIV-1 backbone, and subsequently used to generate virus stocks as described previously (8, 14). Multiple independent bulk and single genome amplified PCRs were pooled to adequately sample the diverse Env quasispecies present in the chronically infected mothers (8, 14). Reference panel Envs were amplified from the panel of global HIV-1 clones (HIV reagent program-12670). The reference Env ectodomains were amplified using forward primer (Env-IF, AGAAAGAGCAGAAGACAGTGGCAATGA) and reverse primer (Env-Ecto, AAGCCTCCTACTATCATTAT) with previously described PCR conditions (13, 14). HIV-1–amplified products were placed in a HIV-1 subtype B NL4-3 backbone using yeast gap-repair homologous recombination methodology as described previously (51, 52). HIV-1 accessory proteins, Vpu and Nef, affect ADCC susceptibility (53). All Envs were incorporated upstream of the Nef protein start site, and, thus, there was no disruption of the ORF. Recombinant clones with incorporated reference panel Envs and maternal Envs with available sequence were confirmed as having Vpu sequences without missense or nonsense mutations. Plasmids containing the reference panel Env within NL4-3 were rescued from yeast, and larger quantities were generated from transformed TOP10 electrocompetent *E*. *coli* cells (Thermo Fisher Scientific). HEK293T cells were transfected with 1–3 μg/μL of the Env containing plasmid, 1 μg/μL of helper plasmid, 94 μL DMEM, and 9 μL polyethylenimine (51, 52). Transfected cells were cultured at 37°C for 48 hours. Supernatants were filtered through a 0.45 μm pore to remove cellular debris before storing at –80°C. HEK293T virus was passaged in CD4^+^ T cells for a maximum of 7 days. Supernatant was filtered through a 0.45 μm pore upon harvest and stored at –80°C. Viral titers were determined on TZM-bl cells in the presence of 10 μg/mL DEAE-dextran (Thermo Fisher Scientific). We were able to generate virus stock containing replication-competent viruses incorporating the following global panel HIV-1 clones (398F1, subtype A; TRO11 and X2278, subtype B; CE1176 and 25710, subtype C; 246F3 AC circulating recombinant form; BJOX2000, CRF07_BC; CNE55 and CNE8, CRF01_AE; and X1632, subtype G). We were not able to generate replication-competent recombinant strains from CH119 (subtype CRF07_BC) or CE0217 (subtype C).

### ADCC assay.

All plasma samples were heat inactivated for 1 hour at 56°C. We estimated the BM-isolated IgG equivalent to the level present in a specified volume of BM supernatants. A 1:50 plasma dilution or equivalent BM IgG present in 1:50 dilution of BM supernatant was used for the ADCC_BP_ assessments. The maternal pool consisted of plasma combined from 10 different BAN women. Autologous Envs were not successfully isolated from these women, and, thus, they did not contribute TM or NTM maternal variants. The bnAb pool contained equivalent amounts of 4 previously described bnAbs (VRC01, PGT121, PG16, and 10E8) (17–20). ADCC sensitivity of the maternal strains was assessed against 6 two-fold dilutions of maternal pool (starting at 1:50 dilution) and bnAbs (starting at 40 μg/mL). All ADCC assays were performed as previously described (8, 13), in triplicate, and a minimum of 2 independent times. The target and effector cells were based on MT4-CCR5-GFP (provided by Paul Bieniasz, the Rockefeller University, New York, New York, USA) and CD16^+^KHYG-1 (provided by David Evans, University of Wisconsin, Madison, Wisconsin, USA) cell lines, respectively (13, 54, 55). Briefly, MT4-CCR5-Luc cells were infected with the virus stock of interest by spinoculating at 1200*g* for 90 minutes before resting cells for 30 minutes at 37°C. Cells were then washed with PBS before incubating for approximately 72 hours at 37°C. After incubation, if RLUs in approximately 1 × 10^5^ infected cells were at least 10-fold over background, then infected cells were used in the ADCC assay. Background RLUs were in unexposed cultured MT4-CCR5-Luc cells. Approximately 1 × 10^5^ infected cells were incubated with Ab source for 20 minutes at 37°C in a 96-well plate (Corning, 3610). After incubation, 5 × 10^5^ CD16^+^KHYG-1 cells were added to each well. After 24 hours, luciferase levels were determined using Bright-Glo (Promega E2650). Differences between RLUs in the presence as compared with the absence of the Ab source were used to determine the percent of ADCC. Background RLUs in uninfected MT4-CCR5-Luc cells and CD16^+^KHYG-1 cells were subtracted from all wells. Serial dilution curves were used to calculate the AUC. The killing capacity of the CD16^+^KHYG-1 cells was assessed each time for every independent ADCC assay by assessing the ability of pooled HIVIG to mediate ADCC against HIV-1 NL43-infected cells.

### Neutralization assay.

Virus neutralization was assessed using the standard HIV-1 TZM-bl assay (56). Briefly, approximately 1000 infectious virus-incorporating maternal Envs were incubated with 6 two-fold dilutions of maternal pool (starting at 1:50 dilution) at 37°C in a total volume of 50 μL for 1 hour at 37°C. Approximately 1E5 TZM-bl cells with 10 μg/mL DEAE-dextran were added to each well after this incubation. After 48 hours, infection levels were determined using Bright-Glo (Promega E2650). Differences between RLUs in the presence of Ab or plasma and growth medium alone were calculated as the percentage of neutralization. Background RLUs in wells with TZM-bl cells alone were subtracted from all wells.

### Human isotyping assay.

All maternal plasma, infant plasma, and BM supernatant was tested using the Bio-Rad Bio-Plex Pro Human Isotyping Assays (171A3100M) to quantify IgG1, IgG2, IgG3, IgG4, IgA, and IgM per manufacturer’s protocol. Samples were tested at a 1:40,000 dilution, and data were acquired on the MAGPIX through access from the Boston University Analytical Core. Total IgG was determined from summing quantities of IgG1, IgG2, IgG3, and IgG4.

### Statistics.

All normally distributed and nonparametric comparisons used an unpaired 2-sided *t* test with Welch’s correction and Mann-Whitney *U* test, respectively. All correlations were assessed using Spearman’s statistic. Multivariate logistic regression analysis was conducted with the transmission status as the dependent variable. Predictors included the various ADCC_BP_ quantifications, or ADCC_AUC_ (maternal pool), ADCC_AUC_ (bnAb pool), or the combined statistics. The covariates of interest included maternal plasma virus level, absolute CD4^+^ T cell count, and days from birth to sample collection. Multivariable linear regression analysis had ADCC_BP_ as the dependent variable. Independent predictors included maternal plasma virus level, absolute CD4^+^ T cell count, days from birth to sample collection, and various Ig levels. Clinical adverse events were graded by the BAN study investigators prior to our sample evaluations and according to toxicity tables from the Division of AIDS at the National Institute of Allergy and Infectious Diseases, NIH. For the Kaplan-Meier event curves and log-rank (Mantel-Cox) analysis, ADCC_AUC_ was dichotomized as high (ADCC_BP_ or ADCC_AUC_ ≥ cohort median) versus low (ADCC_BP_ or ADCC_AUC_ < cohort median). This analysis was stratified by HIV status of the infant. Statistical analysis was done using Stata v17 and GraphPad Prism (Version 8.0). All *P* values are based on 2-sided tests. *P* values of less than 0.05 were considered statistically significant.

### Study approval.

The BAN study was approved by the Malawi National Health Science Research Committee (Lilongwe, Malawi) and the institutional review boards at the University of North Carolina (Chapel Hill, North Carolina, USA), the US Centers for Disease Control and Prevention (Atlanta, Georgia, USA), and Boston University (Boston, Massachusetts, USA). All women provided written informed consent for themselves as well as on behalf of their infants.

## Author contributions

MS conceived and designed the study. AST, CC, YM, and JEI conducted the experiments. ACE and APK provided clinical samples, data, and critical input. MS and AST analyzed the data and conducted the statistical analyses. MS wrote the manuscript with editorial assistance from the coauthors.

## Supplementary Material

Supplemental data

## Version 1 03/24/2022

In-Press Preview

## Version 2 05/09/2022

Electronic publication
