# Supplementary material for: Antibody-dependent cellular cytotoxicity responses and susceptibility influence HIV-1 mother-to-child transmission
Source: JCI Insight. 2022 May 9;7(9):e159435. doi: 10.1172/jci.insight.159435 (PMC9090239; doi:10.1172/jci.insight.159435)
Supplement: Supplemental data [file jciinsight-7-159435-s070.pdf]

Supplementary Table 1. Demographics of matched transmitting and non-transmitting mother infant pairs.

	**TM/HEI**	**NTM/HEU**
**Number of dyads**	21	42
**Median mother age in years (range)**	26 (18-36)	25 (17-36)
**Median mother $\log_{10}$ plasma virus level (range)**	4.78 (3.32-5.80)	4.67 (1.59-5.99)
**Median mother CD4 T cells (cells / ml) (range)**	336 (210-1092)	339 (240-1145)
**Median # of days from birth to sample analyzed (range)**	43 (1-256)	42.5 (12-297)
**Infant age in days at 1st positive HIV-1 PCR test**	83 (42-293)	N/A
**Infant # females (%)**	6 (28.6)	16 (38.1)

TM: transmitting mother; HEI: HIV-1 exposed infected; NTM: non-transmitting mother; HEU: HIV-1 exposed uninfected; N/A: Not applicable

For infant ID 99, there was insufficient sample for analysis of ADCC breadth and potency

Three mother samples were collected within 7 days after delivery. In these 3 cases, IgG was isolated from the sample for the neutralization and ADCC analysis.

One infant sample was collected within 7 days after birth. In this case, IgG was isolated from the sample for the neutralization and ADCC assay.

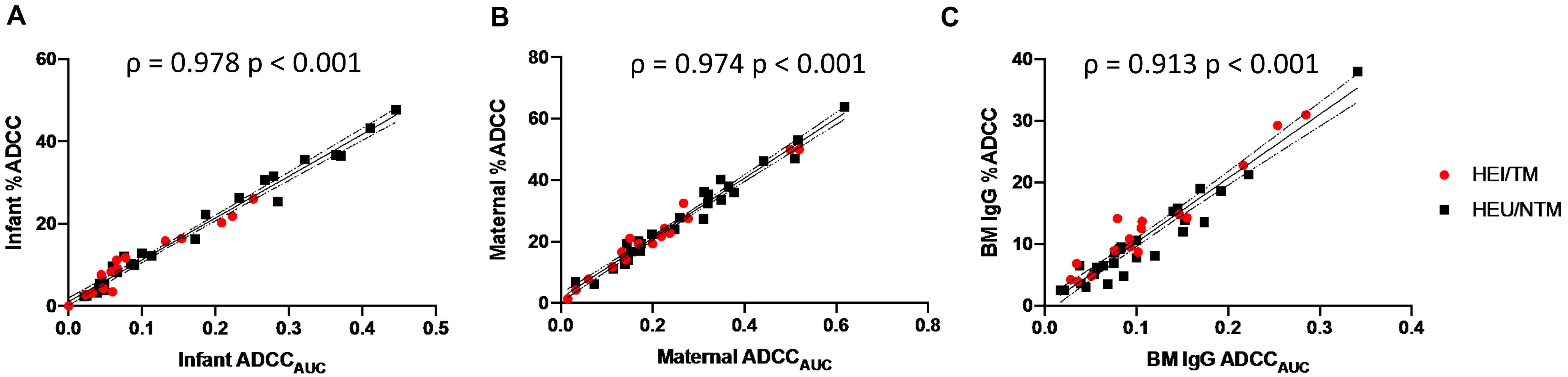

Supplementary Figure 1. Area under the curve (AUC) and Percent ADCC. ADCC measurements using area under the curve ($ADCC_{AUC}$) were compared to measurements of percent ADCC at the top dilution tested (1:50) in infant plasma (A), maternal plasma (B), and BM IgG (C). The red and black dots indicate TMs/ HEI and NTMs/ HEU infants respectively. Correlations were assessed using Spearman's statistic. Line indicates linear regression fit with 95% confidence interval.

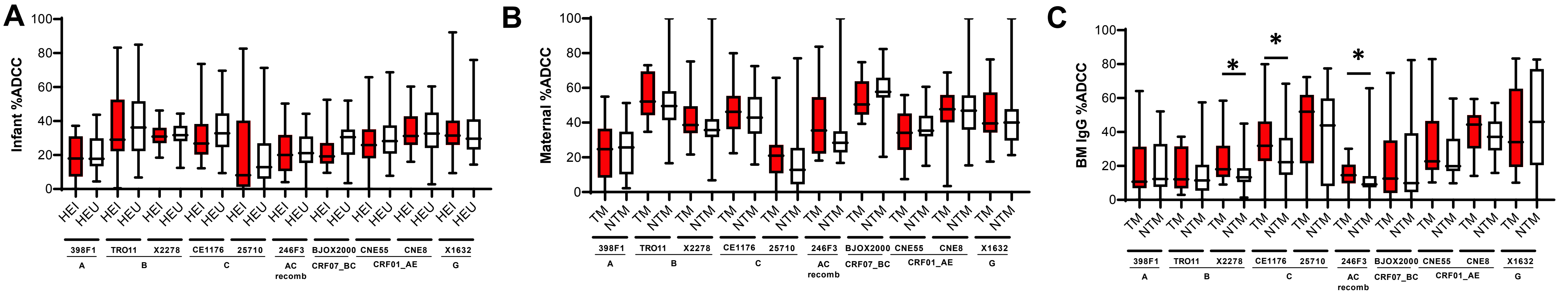

Supplementary Figure 2. Percent ADCC against 10 different Env variants. Percent ADCC measurements at the top dilution tested (1:50) in infant plasma (A), maternal plasma (B), and BM IgG (C) against 10 different variants. The red and black dots indicate TMs/ HEI and NTMs/ HEU respectively. The virus Env ID and subtype are identified on the bottom. Comparisons were done using Wilcoxon rank-sum test. * indicates $p < 0.05$ without accounting for multiple comparisons.

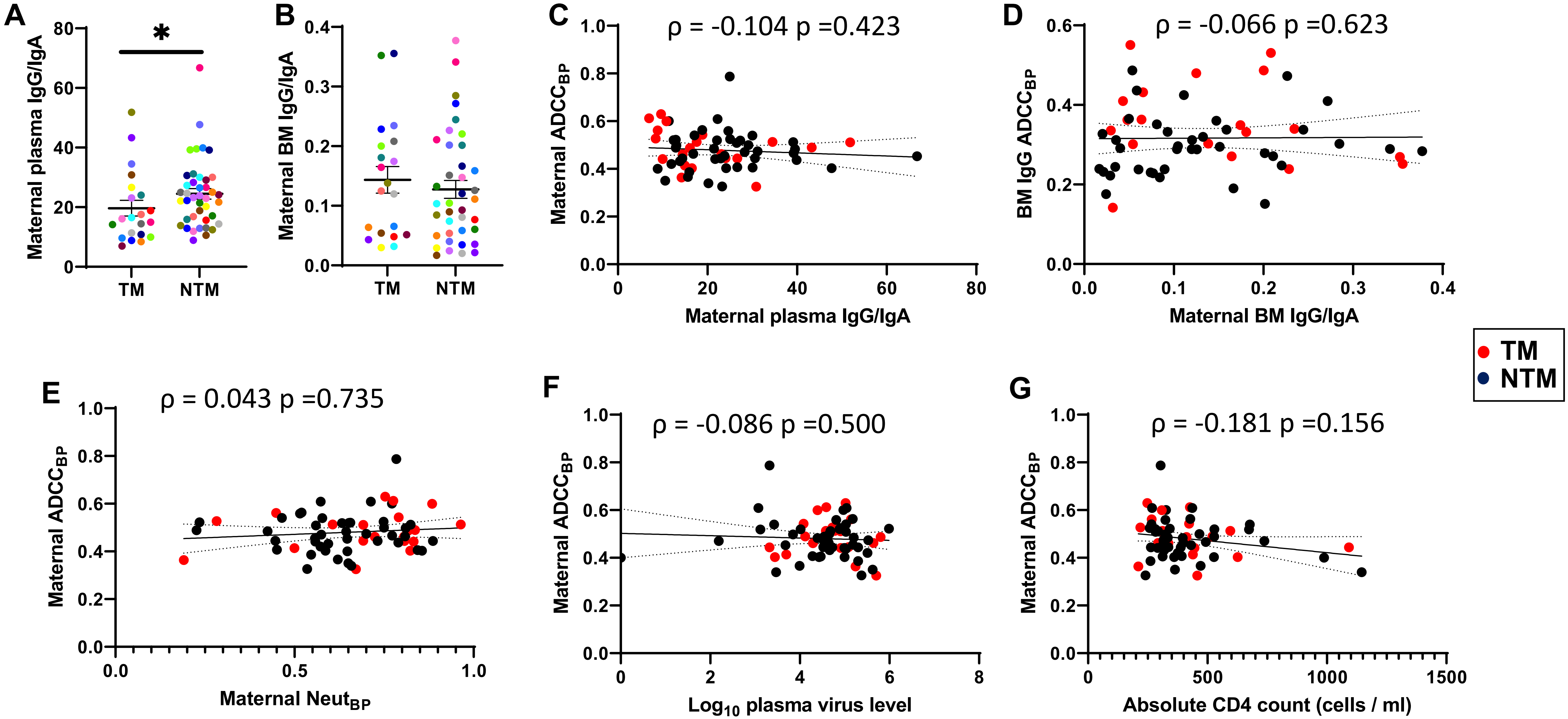

Supplementary Figure 3. Maternal IgG/IgA ratio and correlations among maternal factors. IgG/IgA ratios in TMs and NTMs plasma (A) and breastmilk (BM) (B). Colors indicate matched pairs. * indicates $p \leq 0.05$. Correlations (C – G) among maternal characteristics denoted on the x and y-axis. The red and black dots indicate TMs and NTMs respectively. Correlations were assessed using Spearman's statistic. Line indicates linear regression fit with 95% confidence interval.
